# From simple to supercomplex: mitochondrial genomes of euglenozoan protists

**DOI:** 10.12688/f1000research.8040.2

**Published:** 2016-11-29

**Authors:** Drahomíra Faktorová, Eva Dobáková, Priscila Peña-Diaz, Julius Lukeš

**Affiliations:** 1 Institute of Parasitology, Biology Centre, Czech Academy of Sciences, České Budějovice (Budweis), Czech Republic; 2 Faculty of Sciences, University of South Bohemia, České Budějovice (Budweis), Czech Republic; 3 Departments of Biochemistry and Genetics, Faculty of Natural Sciences, Comenius Universtity, Bratislava, Slovakia; 4 Canadian Institute for Adavanced Research, Toronto, Ontario, Canada

**Keywords:** mitochondria, euglenozoa, mitochondrial genome

## Abstract

Mitochondria are double membrane organelles of endosymbiotic origin, best known for constituting the centre of energetics of a eukaryotic cell. They contain their own mitochondrial genome, which as a consequence of gradual reduction during evolution typically contains less than two dozens of genes. In this review, we highlight the extremely diverse architecture of mitochondrial genomes and mechanisms of gene expression between the three sister groups constituting the phylum Euglenozoa - Euglenida, Diplonemea and Kinetoplastea. The earliest diverging euglenids possess a simplified mitochondrial genome and a conventional gene expression, whereas both are highly complex in the two other groups. The expression of their mitochondrial-encoded proteins requires extensive post-transcriptional modifications guided by complex protein machineries and multiple small RNA molecules. Moreover, the least studied diplonemids, which have been recently discovered as a highly abundant component of the world ocean plankton, possess one of the most complicated mitochondrial genome organisations known to date.

## Introduction

**Figure 1.**
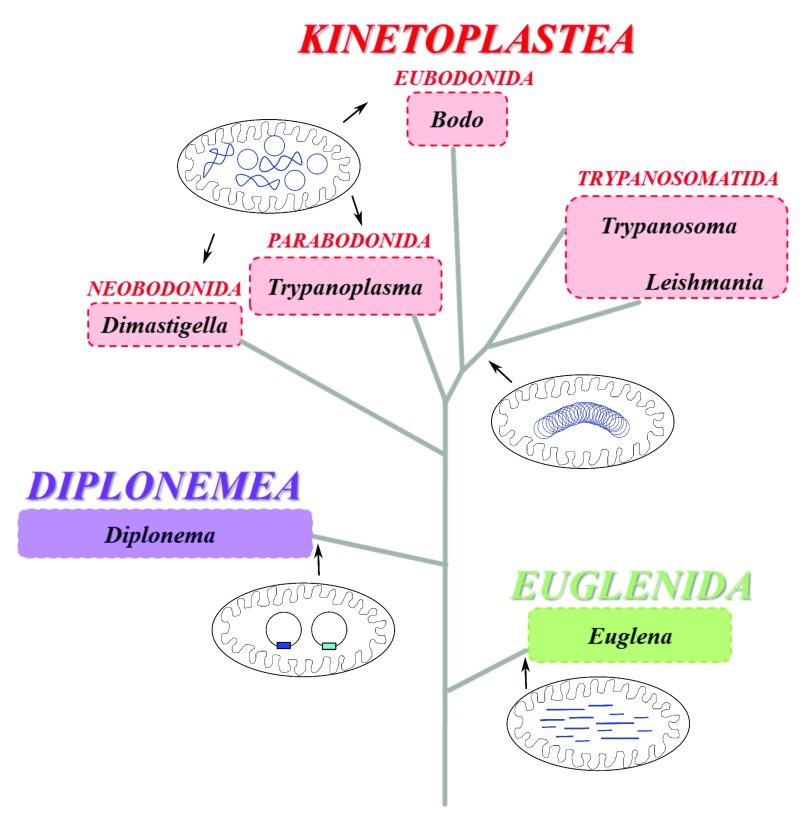
Schematic phylogenetic tree of representative genera of Euglenozoa depicting the organization of their mitochondrial genomes. The scheme is based on Adl *et al.* ^1^ (2012). Different organization of their mitochondrial genomes (in blue) is shown for the three major lineages: Kinetoplastea (in red), Diplonemea (in purple), and Euglenida (in green). Whereas Euglenida possess an array of linear mitochondrial DNA molecules of variable length, Diplonemea and Kinetoplastida contain in their organelle circular DNA molecules in different arrangements. In Diplonemea, circular molecules of two sizes are non-catenated and supercoiled. The kinetoplast DNA of Eubodonida, Parabodonida, and Neobodonida is composed of numerous free, non-catenated relaxed or supercoiled DNA circles, whereas in Trypanosomatida it is constituted of thousands of relaxed circles, mutually interlocked into a single giant network composed of interlocked maxicircles and minicircles that together with proteins are packaged into a single compact disk.

The phylum Euglenozoa, which is part of the supergroup Excavata that significantly diverged from other eukaryotic lineages, is composed of three geographically ubiquitous groups of flagellated protists: Euglenida, Diplonemea, and Kinetoplastea (the fourth group, Symbiontida, has no molecular data available and thus will not be discussed here) ^1^. The well-known representatives of these groups are *Euglena* for euglenids and *Trypanosoma* for kinetoplastids, whereas diplonemids are very poorly known. Although the Euglenozoa is a stable and highly supported group, mutual phylogenetic relationships among these three groups are not yet fully resolved, and euglenids likely constitute the earliest offshoot ( Figure 1) ^1– 3^.

Euglenozoans have several common morphological features, such as subpellicular microtubules and a single flagellum or two heterodynamic flagella, protruding from an anterior pocket. Their lifestyles vary greatly, ranging from the free-living photosynthetic euglenids to intra- and extracellular parasites of plants, insects, and mammals, including humans. We do not yet know the predominant lifestyle of diplonemids, a group that recently came into the spotlight thanks to the Tara Oceans expedition, which revealed their global presence and extreme abundance in the world ocean. Indeed, diplonemids may comprise the sixth most abundant and third most species-rich group of marine eukaryotes ^4, 5^. The euglenids and diplonemids display different modes of nutrition; the former are characterized by photoautotrophy, whereas the latter are likely phagotrophs, osmotrophs, or parasites or a combination of these. The predominantly parasitic kinetoplastids make use of the carbon sources provided by their hosts ^6, 7^.

A hallmark feature of euglenozoans is a single large mitochondrion, frequently reticulated and displaying cristae with a discoid structure ^1, 8^. Like all mitochondria of aerobic protists, this organelle contains mitochondrial DNA (mtDNA) ^7^. Although as vestigial as mtDNAs of other eukaryotes, this organellar genome evolved in euglenozoan protists into a stunning variety of structures and organizations, as described in more detail below. With the advent of affordable high-throughput sequencing, thousands of mt genomes are being assembled and annotated. However, their selection remains strongly skewed toward the metazoans, which mostly harbor standard, highly reduced, and streamlined mt genomes ^9^. Yet the majority of extant eukaryotic diversity is constituted by protists ^10^, of which only a very small fraction has their mtDNA characterized. Still, the available mt genomes of protists show a range of bizarre gene arrangements, modes of organization, and complex post-transcriptional maturations ^11, 12^. Hence, it does not come as a surprise that some authors consider further sequencing of mt genomes of metazoans as superfluous and non-informative but that at the same time they call for focusing efforts onto the organellar genomes of hitherto-neglected protist groups ^12^.

## Mitochondrial genome architecture and gene content

**Figure 2.**
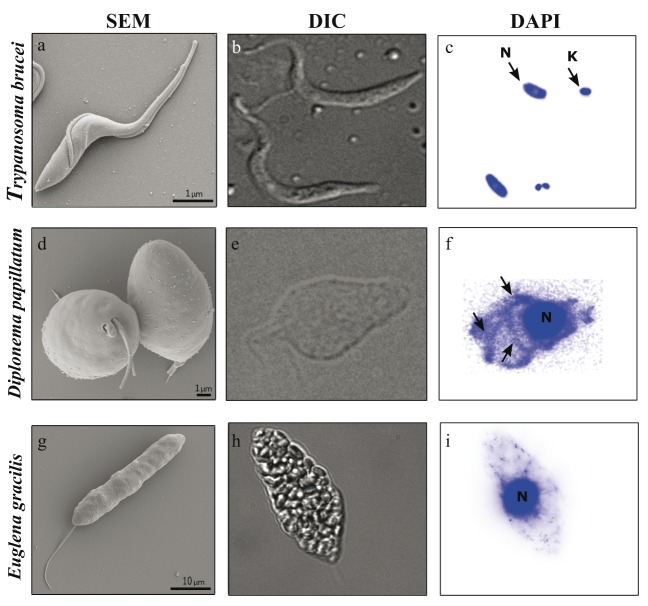
Morphology of representatives of Kinetoplastea ( *Trypanosoma brucei*), Diplonemea ( *Diplonema papillatum*), and Euglenida ( *Euglena gracilis*). Scanning electron microscopy (SEM) ( **a**, **d**, **g**) and differential interference contrast (DIC) ( **b**, **e**, **h**) reveal cell morphology, whereas 4′,6-diamidino-2-phenylindole (DAPI) staining provides information about the amount and distribution of mitochondrial DNA ( **c**, **f**, **i**). ( **c**) *Trypanosoma* with distinct nucleus (N) and kinetoplast (K). ( **f**) In *Diplonema*, arrows point to large amounts of mitochondrial DNA meandering through the cell. Scale bars = 1 μm ( **a**, **d**) and 10 μm ( **g**).

Standard mt genomes are usually represented by a circular or linear DNA molecule encoding an average of fewer than two dozen genes ranging from 2 to 66 proteins in *Chromera velia* and *Andalucia godoyi*, respectively ^13, 14^. Although euglenozoans harbor a low number of genes in their mt genomes, they developed an extremely variable genome architecture. In euglenids and diplonemids, mtDNA seems to be evenly distributed throughout the lumen of the organelle ^8, 15^, whereas in kinetoplastids, the picture is more complex ( Figure 2). In the obligatory parasitic trypanosomatids mtDNA is invariably compacted into a single disk-shaped structure of concatenated DNA termed the kinetoplast DNA (kDNA), the free-living or commensalic bodonids have their kDNA distributed either evenly or in foci in the mt lumen ^16^.

**Figure 3.**
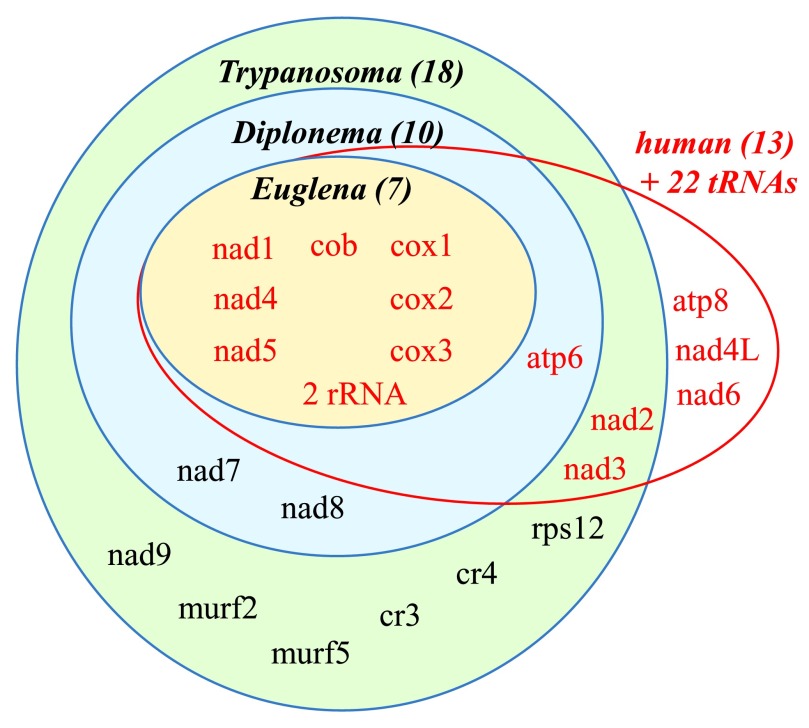
Schematic representation of the gene content of mitochondrial genomes of representative euglenozoans and human. The number of protein-coding genes is shown in parentheses next to selected euglenozoan flagellates. The number of protein-coding genes found in *Euglena* (orange oval), *Diplonema* (blue oval), and *Trypanosoma* is depicted (green oval). In comparison, human mitochondrial genome encodes 13 proteins (in red). All euglenozoans most likely contain small subunit and large subunit mito-rRNAs that in *Diplonema* and *Euglena* are likely to be highly divergent.

The best-studied kDNA is that of the human pathogen and model organism *Trypanosoma brucei* (for current reviews, see 17– 19). It is composed of thousands of DNA circles mutually interlocked into a single network ( Figure 2) that is densely packed into a disk-shaped structure located close to the basal body of the flagellum. The kDNA network of *T. brucei* and related flagellates is formed of dozens of maxicircles, each about 20 kb long, and of approximately 5,000 uniformly sized (~1.0 kb) minicircles ^20^. The maxicircle is composed of a single conserved region, which contains all protein-coding and rRNA genes, and a shorter variable region of species-specific size and sequence, which probably plays a role in replication ^21– 23^. The conserved region carries 18 protein-coding genes, mostly subunits of respiratory complexes (complex I: *nad1*, *nad2*, *nad3*, *nad4*, *nad5*, *nad7*, *nad8*, and *nad9*; complex III: *cob*; complex IV: *cox1*, *cox2*, and *cox3*; complex V: *atp6*), one ribosomal protein ( *rps12*), small and large mito-rRNA genes ( *12S* and *9S*), and four open reading frames of unknown function ( *MURF2*, *MURF5*, *cr3*, and *cr4*) ( Figure 3) ^24, 25^. Each minicircle codes for three to five small guide RNA (gRNA) genes, accounting for a total coding capacity of the kDNA of approximately 1,200 different gRNAs within approximately 250 distinct minicircle classes ^26^. The gRNAs provide information for post-transcriptional RNA editing in the form of multiple insertions and deletions of uridine residues into the maxicircle-derived mRNAs (for recent reviews, see 19, 27, 28). The kDNA does not encode tRNA genes, and hence all tRNA molecules must be imported from the cytosol ^29, 30^.

The mtDNA of the diplonemid *Diplonema papillatum *is composed of numerous free DNA circles ( Figure 1) with a total size of about 600 kb ^31^. The circles fall into two classes that are distinguished by their size (6 or 7 kb) and by the sequence of their non-coding regions that makes up about 95% of the circle. Each circle also carries a single cassette composed of a piece of a gene (gene module) that is flanked on both sides by a unique sequence on average 50 bp in length ^31– 34^. Although the genes are uniquely fragmented, the gene content of *Diplonema *is rather standard and similar to that of kDNA of the kinetoplastid flagellates, as it specifies subunits of respiratory complexes (complex I: *nad1*, *4*, *5*, *7*, and *8*; complex III: *cob*; complex IV: *cox1-3*; complex V: *atp6*) and large subunit (LSU) mito-rRNA ^31^. The small subunit (SSU) mito-rRNA has been identified only very recently because its sequence is extremely diverged, which has made its identification challenging ^34, 35^. So far, all identified mt-encoded genes of *Diplonema *are fragmented, hence must be uniquely *trans*-spliced into fully translatable mRNAs by a hitherto-unknown mechanism.

The mtDNA of *Euglena*, the model euglenid, is surprisingly simple when compared with the highly complex genomes of its sister groups. Indeed, the genome is streamlined in terms of both its architecture and gene content. The mtDNA of *Euglena* is represented by a pool of recombination-prone short linear molecules containing repeats, pieces of non-functional gene fragments, and full-length gene copies, which ensure the production of functional proteins. The set of only three protein-coding genes ( *cox1-3*) ^36, 37^ was recently complemented by four intact genes encoding additional subunits of the respiratory chain ( *nad1*, *4*, and *5* and *cob*) ^38^. The genes encoding subunits of complex V and ribosomal proteins are missing. The SSU and LSU of mito-rRNA are likely split into two fragments each (SSU-R, SSU-L and LSU-R, LSU-L). However, only the SSU-R fragment has been identified to date and this is most likely due to the same reasons as in the case of *Diplonema* LSU, namely the extreme divergence of the corresponding mito-rRNAs ^36, 38^.

## Mechanisms of mitochondrial gene expression

**Figure 4.**
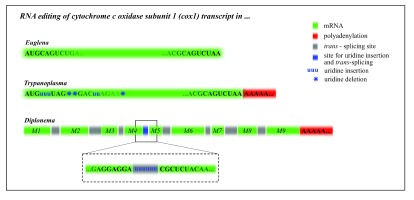
The comparison of RNA processing of the cox1 transcript in representative euglenozoans. In contrast to *Trypanoplasma borreli* and *Diplonema papillatum*, the cytochrome *c* oxidase subunit 1 (cox1) transcript of *Euglena gracilis* (in green) does not undergo RNA editing, splicing, or polyadenylation prior to its translation. In *Trypanoplasma*, cox1 undergoes RNA editing in the form of numerous uridine insertions (small blue “u”) and deletions (blue star), followed by polyadenylation (in red). The *Diplonema* cox1 transcript is formed by *trans*-splicing of nine small fragments called modules M1 thru M9 (in green), which is accompanied by the insertion of six uridines between the modules M4 and M5. Finally, the transcript is polyadenylated and translated on mitochondrial ribosomes.

In all euglenozoans, mtDNA is transcribed into polycistrons, which undergo endonucleolytic cleavage and further editing or processing (or both) into translatable mRNAs ( Figure 4). In kinetoplastids, the majority of mt-encoded genes exist in a cryptic form, as their corresponding transcripts have to undergo extensive post-transcriptional RNA editing of the uridine insertion/deletion type (for recent reviews, see 19, 27, 28). In *Diplonema*, only a few insertions of blocks of uridines have been documented initially, but the recent comprehensive count amounts to ~200 ^31, 32, 35^. RNA editing in *Trypanosoma* and related flagellates is extremely complex, as the insertions and deletions of hundreds and dozens of uridines, respectively, are performed by a multitude of gRNAs and several protein complexes that interact in a highly dynamic manner ^28, 39^. Upon the addition of complex poly-U/A tails ^27, 28^ ( Figure 4), the fully edited transcripts are translated on protein-rich and rRNA-poor ribosomes ^40^, but the role of the additional 45S ribosomal subunit of unique protein composition is still unclear ^41^. Likely owing to their extreme hydrophobicity, only a few of the *de novo* synthesized mt proteins have been observed ^42, 43^.

The post-transcriptional processing is very different in *Diplonema*, where fragments of genes transcribed from individual DNA circles are *trans*-spliced ^31, 33^. The genome and mitoproteome of *Diplonema* may eventually shed light on this unique processing, but so far the proteins (and potentially also small RNAs) involved in it remain completely unknown. In any case, it is highly likely that the fully *trans*-spliced mRNAs are *in organello* translated (our unpublished data). Indeed, the recently described simplicity of mt mRNA processing in *Euglena* ^38^ and likely other euglenids is in stunning contrast with the baroque complexity of RNA editing or *trans*-splicing (or both) in kinetoplastids and diplonemids ( Figure 4 and Table 1).

**Table 1.** Architecture and gene content of mitochondrial genomes of representative euglenozoans and humans.

	*Trypanosoma brucei*	*Diplonema* *papillatum*	*Euglena gracilis*	Human
Type of mitochondrial cristae	Discoidal	Discoidal	Discoidal	Tubular
Mode of life	Parasitic	Phagotrophic osmotrophic	Mixotrophic heterotrophic autotrophic	Heterotrophic
Habitat	Insect gut mammalian bloodstream	Marine	Freshwater	Predominantly terrestral
Genome structure	Circular	Circular	Linear	Circular
protein-coding genes	18	10	7*	13
rRNA (SSU/LSU)	+/+	+/+	+/?	+/+
tRNA	-	-	-	22
Chromosome size	Maxicircles: ~20.0 kb Minicircles: ~1.0 kb	Class A: 6.0 kb Class B: 7.0 kb	~1.0 to ~9.0 kb	16.6 kb
Genome copy number	Maxicircles: ~dozens Minicircles: ~5,000	~100	?	~50 to 1×10 ^5^
mRNA polyadenylation	Yes	Yes	No	Yes
*Trans*-splicing	No	Yes	No	No
Uridine insertions	Yes	Yes	No	No
Uridine deletions	Yes	No	No	No
Introns	No	No	No	No

*Seven complete genes, together with multiple gene fragments.

## Why are mitochondrial genomes in Euglenozoa so diverse?

Soon after its discovery, RNA editing in kinetoplastids was explained as a remnant of the RNA world ^44^. A more plausible explanation postulates that gene fragmentation in the euglenozoan last common ancestor was “the seed of future chaos”, leading to the emergence of extremely complex post-transcriptional mechanisms, differing in each sister lineage, yet eventually correcting the scrambling on the post-transcriptional level ^45^. In fact, it was argued that the kinetoplastid RNA editing is a prime example of “irremediable complexity”, a rampant mechanism that does not provide any selective advantage yet fixes the problem ^39, 46^. The recent finding of a mt genome in *Euglena* ^38^ implies that the irreversible scrambling originally implied for the mtDNA of the euglenozoan last common ancestor ^45^ did happen at a later stage in evolution, probably in the predecessor of diplonemids and kinetoplastids. Although despite the available sequence data the mutual relationships among the three euglenozoan lineages remain unresolved, we can predict, on the basis of their mt genomes and transcriptomes, that the mostly free-living photosynthetic euglenids constitute the earliest offshoot of the long euglenozoan branch.
